# Comparison of electrophysiological findings in axonal and demyelinating Guillain-Barre syndrome

**Published:** 2014-07-04

**Authors:** Samira Yadegari, Shahriar Nafissi, Neda Kazemi

**Affiliations:** Department of Neurology, School of Medicine, Tehran University of Medical Sciences, Tehran, Iran

**Keywords:** Guillain-Barre Syndrome, Electrophysiology, Cerebrospinal Fluid, Nerve Conduction Study

## Abstract

**Background: **Incidence and predominant subtype of Guillain-Barre syndrome (GBS) differs geographically. Electrophysiology has an important role in early diagnosis and prediction of prognosis. This study is conducted to determine the frequent subtype of GBS in a large group of patients in Iran and compare nerve conduction studies in axonal and demyelinating forms of GBS.

**Methods:** We retrospectively evaluated the medical records and electrodiagnostic study (EDS) of 121 GBS patients who were managed in our hospital during 11 years. After regarding the exclusion criteria, patients classified as three groups: acute inflammatory demyelinating polyneuropathy (AIDP), acute motor axonal neuropathy (AMAN), and acute motor sensory axonal neuropathy (AMSAN). The most frequent subtype and then electrophysiological characteristic based on the time of EDS and their cerebrospinal fluid (CSF) profile were assessed.

**Results: **Among 70 patients finally included in the study, 67% were men. About 63%, 23%, and 14% had AIDP, AMAN, and AMSAN, respectively. AIDP patients represented a wider range of ages compared with other groups. Higher levels of CSF protein, abnormal late responses and sural sparing were more frequent in AIDP subtype. Five AMSAN patients also revealed sural sparing. Conduction block (CB) was observed in one AMAN patient. Prolonged F-wave latency was observed only in AIDP cases. CB and inexcitable sensory nerves were more frequent after 2 weeks, but reduced F-wave persistency was more prominent in the early phase.

**Conclusion: **AIDP was the most frequent subtype. Although the electrophysiology and CSF are important diagnostic tools, classification should not be made based on a distinct finding.

## Introduction

Guillain-Barre syndrome (GBS) is an immune mediated disorder of peripheral nerves which usually presents sporadically^1^ with incidence of 1-2 cases per 100,000 populations.^2^ However, its prevalence may vary in different regions.^3^ Although clinical and laboratory findings have an important role in the diagnosis of GBS, electrodiagnostic study (EDS) is the basis for classification of different subtypes of the disease. Based on electrophysiological findings, GBS has three major subtypes: acute inflammatory demyelinating polyneuropathy (AIDP), acute motor axonal neuropathy (AMAN), and acute motor sensory axonal neuropathy (AMSAN). Predominant subtype of GBS differs according to the geographic area.

EDS also has a crucial role in diagnosis, ruling out of some differential diagnosis like myopathic and motor neuron disorders and confirming the neuropathic nature of GBS.^4^ True and early diagnosis of GBS could impact on its prognosis, as the benefit of immunotherapy is greatest when introduced early, in the first few weeks of disease.^2^ In addition, electrophysiological characteristic, by itself, could guide the clinicians to predict the prognosis of patients with GBS.^2^

Hence, this study is conducted to determine: (1) predominant subtype of GBS, (2) electrophysiological pattern and comparison of characteristics of EDSs in early and late phase of GBS and (3) cerebrospinal fluid (CSF) profile among different electrophysiological subtypes.

## Materials and Methods

We reviewed data from consecutive patients with a confirmed clinical and laboratory diagnosis of GBS who were admitted in our hospital during 11 years. The diagnosis of GBS was made based on Albers and Kelly criteria.^5^ We retrospectively searched electrophysiological and hospital records of the patients in neuromuscular unit from January 1997 to October 2007. Information about age, sex, season of illness, CSF profile, and time from the onset of disease and performing EDS were considered.

EDS including nerve conduction study (NCS) and electromyography were performed for all patients, using surface electrodes and stimulator for NCS. Limb temperature was maintained above 32° C using warmer, if needed. Standard motor and antidromic sensory NCS were performed in at least four motor nerves (Median, ulnar, tibial, and peroneal) and three sensory nerves (Median, ulnar, and sural). In motor nerves, distal latency (DL), amplitude and duration of compound muscle action potential (CMAP), nerve conduction velocity (NCV), conduction block (CB), and temporal dispersion (TD) were evaluated. CB and TD considered according to the definition of American Association of Neuromuscular and Electrodiagnostic consensus.^6^ F-wave minimal latency was measured after supramaximal stimulation of motor nerves and identifying 10 F-waves. H reflex was recorded from soleus after stimulation of tibial nerve. Amplitude of sensory nerve action potential (SNAP), peak latency and NCV was measured in sensory nerves. Sural sparing was defined as normal or relatively preserved sural SNAP compared with at least two abnormal SNAPs in the upper limb.^7^ Abnormality was considered when values were beyond mean ± 2.5 standard deviation of our laboratory control.

After reviewing hospital records of our GBS patients, we excluded normal (n = 5) or near normal (n = 4) electrophysiological findings. One patient had pure sensory involvement (Probably acute sensory axonal neuropathy) which was excluded. In addition, when electrophysiological data were not available in details, patients were excluded from the study. The cases were finally classified into three groups: AIDP, AMAN, or AMSAN. AIDP was diagnosed based on Albers and Kelly criteria.^5^ When there was no evidence of demyelinating criteria, patients were classified as having AMAN. AMSAN was defined as the presence of AMAN pattern in motor nerve studies along with more than 50% reduction of normal SNAP amplitude in two or more sensory nerves.^8^ Then, we evaluated the electrophysiological characteristics of the patients according to the time of NCS after the onset of symptoms, whether it was done in the first 2 weeks (early NCS) or after that (late NCS). The 2 weeks limit was considered in several other studies of GBS.^7^^,^^9^^,^^10^ Statistical analysis was done using SPSS for Windows 17.0 (SPSS Inc., Chicago, IL, USA). We used non-parametric analysis, Kruskal-Wallis test, for quantitative variables and chi-square test for others. P < 0.05 was considered as significant. The study protocol was approved in the Ethical Committee of Shariati Hospital (Tehran University of Medical Sciences, Tehran, Iran), where the research was performed.

## Results

**Figure 1:**
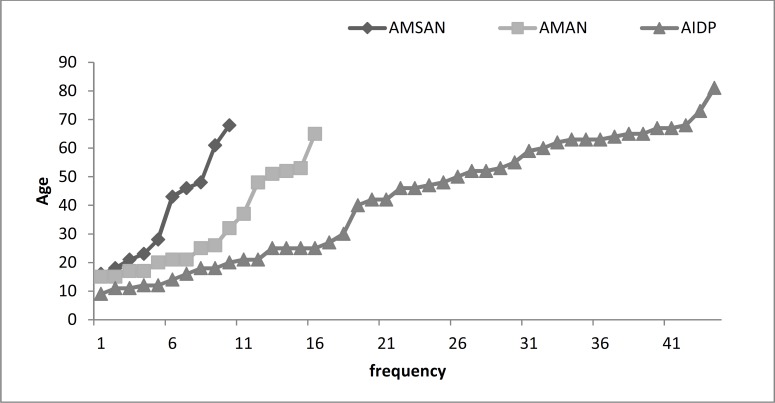
Age distribution among electrophysiological subtypes of Guillain-Barre syndrome

A total of 121 patients was admitted and discharged with the diagnosis of GBS during the period of study. After regarding the exclusion criteria (Above-mentioned), 70 patients were included in the final analysis. Patients were 67% male (n = 47) and the mean age was 39 years. The most common type of GBS was AIDP (63%) followed by AMAN (23%) and AMSAN (14%). There was no significant seasonal clustering among electrophysiological subtypes. In each GBS subtypes, demographic data and CSF findings are summarized in table 1 and age distribution was shown in figure 1. CSF analysis was performed in 54 patients, and albuminocytological dissociation (ACD) was found in 87%. The median and mean of CSF protein did not have a significant difference between three electrophysiological subtypes. Hence, in order to find whether there was a significant difference in the amount of CSF protein we compared the 75% and then 95% percentile of CSF protein in axonal and demyelinating subtypes. CSF protein in none of these percentiles differed significantly between subtypes.

Among 70 GBS patients, NCS was performed in 25 patients in the first 2 weeks of symptom onset and after that period in 45 patients. The mean interval between the onset of symptoms and time of NCS was 9.3 ± 3.1 (range 2-13) and 21.6 ± 9.3 (range 14-60) days in early and late groups, respectively. The results of motor and sensory NCSs based on the time of NCS are detailed in tables 2 and 3. In AIDP patients with early NCS, prominent CMAP amplitude reduction was observed in tibial nerves, while in axonal types, this reduction was the same in upper and lower limbs. In sensory NCS, prominent discrepancy between upper and lower limbs was seen in each electrophysiological subtype based on the time of NCS: in first 2 weeks, SNAP amplitude was comparably reduced in both upper and lower limbs in AIDP, but with progression of time, sural SNAP amplitude showed more preservation than median and ulnar SNAPs. The median of SNAP amplitude remained normal in early and late groups of AMAN, but SNAP amplitudes revealed further reduction in the late group of AMSAN which in contrast with AIDP, was more prominent in sural nerve.

The most frequent inexcitable motor nerve in both early and late groups was peroneal nerve with the same frequency of 20% (Table 4). While, the most frequent inexcitable sensory nerve in patients who underwent NCS in their first 2 weeks was sural (n = 9, 36%) and after 2 weeks was ulnar nerve (n = 23, 56%). As shown in table, inexcitable nerves are clearly more frequent in sensory than motor nerves, especially when NCS was performed late. Overall, inexcitable nerves were present in 85 of 308 (27.6%) and 26 of 182 total examined nerves (14.3%), in AIDP and axonal types of GBS, respectively.

CB and TD were present in 40% and 33% of early NCS in AIDPs. These parameters were more frequent with progression of time after 2 weeks, presented in about half of patients (Table 5). In the first 2 weeks, seven patients represented CB which three of them were in median, two in ulnar, one in peroneal, and one in tibial nerves. One of median CBs was occurred in an AMAN patient. Only one patient showed CB in more than one nerve (Ulnar, peroneal, and tibial) in this time. After 2 weeks, tibial nerve had the most frequent CB (7/14) and CB in more than one nerve was detected in seven patients. Total number of CB and TD based on each studied motor nerve were summarized in table 4.

Table 5 shows other electrophysiological features of GBS patients. Unobtainable H-reflex was the late response with the greatest frequency of abnormality. Abnormal late responses were obviously common in demyelinating rather than axonal GBS. F-response was abnormal in 52.5% of cases. The most frequent abnormality of F-response was prolonged F-wave latency (23%). Unobtainable F was detected in 15.7% of patients (8 patients with AIDP and 3 with AMAN). In cases evaluated in the first 2 weeks, reduced F-wave persistency was more frequently seen than those who underwent NCS after 2 weeks. The frequency of sural sparing was 30%. From a total of 21 patients who showed sural sparing, 76.2% (n = 16) were in AIDP and 23.8% (n = 5) in AMSAN groups, but this difference did not achieve statistical significance; because in the rest of patients who did not develop sural sparing, AIDP was more prevalent again (57%). 

**Table 1:** Demographic and cerebrospinal fluid findings in patients with Guillain-Barre syndrome

**Patients characteristics**	**AIDP, n = 44**	**AMAN, n = 16**	**AMSAN, n = 10**	**P**
Age, mean (range)	46 (9-81)	26 (15-65)	36 (16-68)	0.422
Gender M/F	29/15	13/3	5/5	0.206
CSF protein, mean (range)	120 (15-1130)	97 (32-142)	96 (20-152)	0.263

AIDP: Acute inflammatory demyelinating polyneuropathy; AMAN: Acute motor axonal polyneuropathy; AMSAN: Acute motor sensory axonal polyneuropathy; CSF: Cerebrospinal fluid

**Table 2:** Results of motor nerve conduction studies in patients with Guillain-Barre syndrome^a^

**Nerve**	**Normal control**	**Early** ^b^ **, < 2 weeks**			**Late** ^b^ **, ≥ 2 weeks**		
		**AIDP, n = 15**	**AMAN, n = 8**	**AMSAN, n = 2**	**AIDP, n = 29**	**AMAN, n = 8**	**AMSAN, n = 8**
Median							
CMAP amplitude (mV)	≥ 4.0	2.1 (0-11.0)	2.9 (0-11.0)	3.30 (3.0-4.0)	3.4 (0-10.0)	2.8 (0-9.0)	3.2 (1.0-11.0)
Motor DL (ms)	≤ 4.4	6.4 (3.0-13)	3.7 (3.0-5)	3.10 (3.0-4.0)	7.1 (NA-24.0)	3.9 (3-9.0)	3.6 (3.0-6.0)
NCV (m/s)	≥ 49	47.8 (NA-70.0)	53.0 (NA-60.4)	52.7 (48.0-57.4)	37.8 (NA-55.0)	53.6 (50.0-62)	48.0 (43.0-56.0)
Ulnar							
CMAP amplitude (mV)	≥ 6.0	3.0 (0-6.0)	3.6 (0-5.7)	2.8 (1.3-4.3)	4.0 (0-12.6)	2.2 (0-11.1)	5.6 (0.5-13.8)
Motor DL (ms)	≤ 3.3	4.2 (NA-8.5)	3 (NA-4.2)	3.1 (3-3.2)	4.2 (NA-14.2)	3.1 (NA-3.8)	3.0 (2.2-4.9)
NCV (m/s) (B.e)	≥ 49	45 (NA–63.3)	60 (NA–68)	67.5 (67-68)	43 (NA-75.7)	59.6 (NA-75)	58.5 (49-70)
NCV (m/s) (A.e)	≥ 44	32 (NA-55)	45 (NA-75)	38 (32-44)	38 (NA-69.9)	54 (NA-72.)	54.5 (43-74)
Peroneal							
CMAP amplitude (mV)	≥ 2	1.2 (0-6.1)	0.4 (0-8.8)	0	1.2 (0-11.5)	0.9 (0-3.5)	0.5 (0-3.3)
Motor DL (ms)	≤ 6.5	9.0 (NA-16.2)	5.3 (NA-7.3)	NA	7.5 (NA-16.5)	6.3 (NA-8.3)	4.8 (NA-6.7)
NCV (m/s)	≥ 44	33 (NA-58.7)	44.8 (NA–48)	NA	32.6 (NA-65.2)	44 (NA-59.6)	35 (NA-42)
Tibial							
CMAP amplitude (mV)	≥ 4	0.5 (0-5.3)	1.2 (0.1-6.3)	1.5 (1.4-1.6)	2.5 (0-12.3)	1.5 (0-8.0)	1.5 (0-7.7)
Motor DL (ms)	≤ 5.8	6.7 (NA-14.2)	7.1 (5.1-8.9)	5.05 (5.0-5.1)	6.5 (NA–14.7)	6.7 (NA–8.9)	4.5 (NA-7.5)
NCV (m/s)	≥ 41	27.6 (NA-71)	45 (37.2-47)	45.8 (41.7-50)	28.0 (NA–55.3)	39.4 (NA-46)	39 (NA-46)

AIDP: Acute inflammatory demyelinating polyneuropathy; AMAN: Acute motor axonal polyneuropathy

AMSAN: Acute motor sensory axonal polyneuropathy; NCV: Nerve conduction velocity; DL: Distal latency

CMAP: Compound muscle action potential; B.e: Below elbow, A.e: Above elbow; NA: Not applicable

a Data are presented in median (range).

b Time of nerve conduction study from onset of symptoms.

**Table 3:** Results of sensory nerve conduction studies in patients with Guillain-Barre syndrome^a^

**Nerve**	**Normal ** **control**	**Early** ^a^ **, < 2 weeks**			**Late** ^a^ **, ≥ 2 weeks**		
		**AIDP, n = 15**	**AMAN, n = 8**	**AMSAN, n = 2**	**AIDP, n = 29**	**AMAN, n = 8**	**AMSAN, n = 8**
Median							
Amplitude (µV)	≥ 20	10.1 (0-86)	48.8 (0-74)	41.2 (21.4-61)	0 (0-81)	45.3 (6.5-70.8)	9.6 (0-43)
NCV (ms)	≥ 50	43.2 (NA-67)	53 (NA-62.2)	59.5 (54-56)	NA (NA-63)	55.8 (28.0-62.8)	54.6(NA-66)
Ulnar							
Amplitude (µV)	≥ 17	10.2 (0-50)	36 (16-54)	33 (23.2-42.8)	0 (0-58)	36.5 (20-72.4)	6.5 (0-41)
NCV (ms)	≥ 50	49 (NA-67)	62 (48.6-63)	62.9 (53-72.8)	NA (NA-68)	52.9 (50-58)	49.4 (NA–65)
Sural							
Amplitude (µV)	≥ 6	2.8 (0-16.0)	12.0 (3.2-32.0)	0	5.5 (0-21.6)	13.7 (8.7-22.0)	0 (0-13.9)
NCV (ms)	≥ 40	39 (NA-55)	44 (39-59.4)	NA	38 (NA-61.6)	46 (35.7-49.3)	NA (NA-63)

AMAN: Acute motor axonal polyneuropathy; AMSAN: Acute motor sensory axonal polyneuropathy; NCV: Nerve conduction velocity

NA: Not applicable

a Data are presented in median (range). AIDP: Acute inflammatory demyelinating polyneuropathy

**Table 4:** Motor and sensory nerves electrophysiological characteristics

**Electrophysiological feature**	**Motor nerves**				**Sensory nerves**		
	**Median**	**Ulnar**	**Peroneal**	**Tibial**	**Median**	**Ulnar**	**Sural**
Inexcitable nerves < 2 weeks (n = 25)	1 (4.0)	2 (8.0)	5 (20.0)	2 (8.0)	8 (32.0)	6 (24.0)	9 (36.0)
Inexcitable nerves ≥ 2weeks (n = 45)	1 (2.2)	2 (4.4)	9 (20.0)	7 (15.6)	20 (44.0)	23 (56.0)	16 (36.0)
Conduction block	5 (7.1)	6 (8.5)	7 (10.0)	8 (11.4)	-	-	-
Temporal dispersion	9 (12.8)	3 (4.3)	3 (4.3)	4 (5.7)	-	-	-
Absent F-wave	3 (4.3)	4 (5.7)	15 (21.4)	9 (12.8)	-	-	-

Data in parenthesis are indicative of %

**Table 5:** Electrodiagnostic characteristics based on the time of study in patients with Guillain-Barre syndrome

**Nerve**	**Early** ^a^ **, < 2 weeks**			**Late** ^a^ **, ≥ 2 weeks**		
	**AIDP, n = 15**	**AMAN, n = 8**	**AMSAN, n = 2**	**AIDP, n = 29**	**AMAN, n = 8**	**AMSAN, n = 8**
CB, f (%)	6 (40.0)	1 (12.5)	0	14 (48.3)	0	0
TD, f (%)	5 (33.3)	0	0	14 (48.3)	0	0
Absent F-wave, f (%)	3 (20.0)	1 (12.5)	0	5 (17.2)	2 (25.0)	0
Prolonged F-wave, f (%)	6 (40.0)	0	0	10 (34.5)	0	0
F-wave persistency %, Median (range)	15 (0-60)	70 (0-90)	50 (50-50)	40 (0-100)	80 (0-100)	80 (60-90)
Unobtainable H reflex, f (%)	12 (80.0)	2 (25.0)	0	11 (37.9)	5 (62.5)	2 (25.0)
Sural sparing	5 (33.3)	0	0	11 (37.9)	0	5 (62.5)

AIDP: Acute inflammatory demyelinating polyneuropathy

AMAN: Acute motor axonal polyneuropathy; AMSAN: Acute motor sensory axonal polyneuropathy; CB: Conduction block

TD: Temporal dispersion

a Time of nerve conduction study from onset of symptoms.

## Discussion

GBS is a widely distributed disease throughout the world that affects all ethnic and age groups, but the predominant electrophysiological subtype may differ geographically. Our study showed that AIDP is the major subtype of GBS in Iran. This study was conducted in a tertiary referral center in Tehran and subjects who are referred from different areas could be a fair representative of the whole country. Another study in Northwest of Iran reported demyelination in 60.5%, axonal in 25% and mixed pattern in 14.5% of patients with mean annual incidence of 2.11/100,000 populations.^11^ A study from the neighboring country of Kuwait, in West of Iran, showed that of 41 cases, 61% had demyelinating, 15% axonal, 5% mixed, 5% H reflex abnormality alone, and 5% normal NCS.^12^ Another neighboring country in East of Iran, Pakistan, showed a relative similar pattern of GBS with demyelinating type in 46%, axonal in 31% and unclassifiable in the rest of their 175 cases.^13^ Consequently, the main type of GBS in Middle-east is probably AIDP. On the other hand, along with the predominancy of demyelinating pattern in this part of the world, the axonal variants of GBS seems more prevalent than North America and Europe which include only 5% of GBS^4^ and less common than East of Asia, Japan which have reported AMAN in 45-48% of their GBS.^14^^,^^15^

GBS in our patients was seen in a wide range of age, but mean age of patients was lower in AMAN (2 decades) and AMSAN than AIDP. The tendency of AMAN to ages younger than 40 was observed in other studies.^15^^,^^16^ However, AMAN in children was mostly reported in epidemics in the Northern China.^2^

ACD in CSF analysis showed no significant difference between demyelinating and axonal types of GBS in our study and we could not find a cut-off to splitting these types based on CSF protein. However, the amount of CSF protein in AIDP was higher than axonal variants and very high levels of protein were detected only in demyelinating form. Hence, beside the rise in CSF protein without cell, which may be found in AMAN,^2^ the amount of protein also should be considered in interpretation of CSF finding in GBS. The rise in CSF protein in GBS is attributed to damage of proximal nerve root myelin or axon, which results to release of proteins either myelin sheath-associated markers (Myelin basic protein) or axonal damage markers (Neurofilaments, tau, and anti-ganglioside antibodies) into CSF.^17^

Unexcitable nerves were more common among examined sensory nerves, especially those evaluated after 2 weeks. The reason is probably related to the time that takes Wallerian degeneration occurs, which is longer for sensory than motor nerves and subsequently results in SNAP amplitude reduction to its nadir later than CMAP amplitude.^18^ Lower amplitude of CMAP in the median and tibial nerves in the early phase of AIDP compared to axonal variants may be due to proximal CBs. With progression of time, CMAP amplitudes were decreased more in axonal types which reflected axonal degeneration.

Sural sparing is a hallmark of demyelination and found in 36.3% of AIDPs in our study. Five AMSAN patients also revealed sural sparing. On the other look, in GBS patients who had sural sparing, 76% were AIDP. Our findings suggest that sural sparing may not be so sensitive to detection of demyelination, but in its presence, AIDP is more likely than AMSAN. Gupta et al. found a frequency of 34.5% in the 2^nd^ week after symptom onset of GBS in India.^16^ In another study, sural sparing showed sensitivity and specificity of 48% and 98%, respectively, for acquired demyelination when compared with the critical illness neuropathy.^19^

CB was observed in one AMAN patient who was evaluated in the first 2 weeks, in wrist- elbow segment of the median nerve. Reversible conduction failure at the axolemma of the Ranvier node was suggested as a transitional phase in pathophysiology of axonal GBS, especially in early stage of the disease.^20^^,^^21^ Therefore, although CB is considered in most criteria as a hallmark of demyelination, but there are exceptions. In this challenging situation, serial NCS is the best way in order to prevent a misdiagnosis of demyelination. Next NCS had not been done for this patient. However, he had no more criteria required for diagnosis of demyelination other than CB in one nerve. Between motor nerves, CB was more frequent in lower limbs especially tibial nerve, which could be related to long length of these nerves.

Unobtainable H reflex was the most common finding in our AIDPs, which could reflect its high sensitivity, but since some patients with axonal GBS also represented this feature (Table 5), specificity is probably low. In previous studies, a sensitivity of 95% was reported for absent H reflex in AIDP, in spite of its low specificity (33%).^19^ Unlike H reflex, absent or prolonged F response with relatively preserved distal CMAP amplitude might be more specific for demyelination.^19^ In our study, prolonged F-wave latency was observed only in AIDP cases. In addition, median of F-wave persistency was prominently reduced in this subtype of GBS. Among studied motor nerves, absent F-wave was more frequent in peroneal nerve probably due to low amplitude CMAPs in this nerve.

## Conclusion

Our study showed that the most common type of GBS in Iran was AIDP. Higher levels of CSF protein, unobtainable H-reflex and F-response, sural sparing and unexcitable nerves were more frequent in this subtype. With progression of time, CB and TD in AIDPs, SNAP amplitude reduction in AMSANs and sensory unexcitable nerves became more frequent. In addition, although the rise in CSF protein and sural sparing are more frequent in demyelinating variant, but these may not have enough sensitivity to discriminate AIDP from axonal subtypes. Hence, the diagnosis of GBS and defining its subtypes should not be made based on a single finding and clinical features, CSF profile and electrodiagnostic evaluation should be considered together.

## Notes

**How to cite this article: **Yadegari S, Nafissi Sh, Kazemi N. Comparison of electrophysiological findings in axonal and demyelinating Guillain-Barre syndrome. Iran J Neurol 2014; 13(3): 138-43.
